# Tetra­aqua­bis­[2-(pyridin-4-yl-κ*N*)pyrimidine-5-carboxyl­ato]zinc

**DOI:** 10.1107/S160053681204411X

**Published:** 2012-10-31

**Authors:** Rupam Sen, Dasarath Mal, Paula Brandao, Zhi Lin

**Affiliations:** a Department of Chemistry, CICECO, University of Aveiro, 3810-193 Portugal

## Abstract

In the title complex, [Zn(C_10_H_6_N_3_O_2_)_2_(H_2_O)_4_], the Zn^II^ ion lies on an inversion center and is coordinated in a slightly distorted octa­hedral geometry by two N atoms from two 2-(pyridin-4-yl)pyrimidine-5-carboxyl­ate ligands and four water mol­ecules. In the symmetry-unique part of the mol­ecule, the pyridine and pyrimidine rings form a dihedral angle of 7.0 (1)°. In the crystal, the coordinating water mol­ecules act as donor groups and carboxyl­ate O atoms act as acceptors in O—H⋯O hydrogen bonds, forming a three-dimensional network.

## Related literature

For a general background to supra­molecular chemistry, see: Collet *et al.* (1996 ▶). For general syntheses and applications of MOFs, see: Sen *et al.* (2012 ▶); Saha *et al.* (2012 ▶). For a related structure, see: Piao & Xuan (2011 ▶).
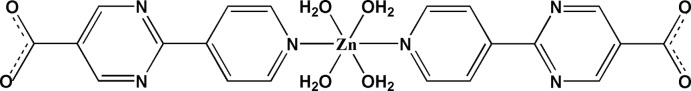


## Experimental

### 

#### Crystal data

[Zn(C_10_H_6_N_3_O_2_)_2_(H_2_O)_4_]
*M* *_r_* = 537.79
Triclinic, 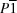

*a* = 6.2764 (7) Å
*b* = 6.9208 (7) Å
*c* = 12.7810 (17) Å
α = 99.676 (7)°
β = 92.638 (7)°
γ = 112.639 (5)°
*V* = 501.36 (10) Å^3^
*Z* = 1
Mo *K*α radiation
μ = 1.29 mm^−1^
*T* = 150 K
0.26 × 0.20 × 0.04 mm

#### Data collection

Bruker SMART CCD diffractometer
Absorption correction: multi-scan (*SADABS*; Bruker, 2008 ▶) *T*_min_ = 0.730, *T*_max_ = 0.950
8143 measured reflections
2184 independent reflections
2030 reflections with *I* > 2σ(*I*)
*R*_int_ = 0.032

#### Refinement

*R*[*F* ^2^ > 2σ(*F* ^2^)] = 0.033
*wR*(*F* ^2^) = 0.089
*S* = 1.14
2184 reflections
176 parameters
4 restraints
H atoms treated by a mixture of independent and constrained refinement
Δρ_max_ = 1.05 e Å^−3^
Δρ_min_ = −0.61 e Å^−3^

### 

Data collection: *SMART* (Bruker, 2008 ▶); cell refinement: *SAINT* (Bruker, 2008 ▶); data reduction: *SAINT*; program(s) used to solve structure: *SHELXS97* (Sheldrick, 2008 ▶); program(s) used to refine structure: *SHELXL97* (Sheldrick, 2008 ▶); molecular graphics: *Mercury* (Macrae *et al.*, 2006 ▶); software used to prepare material for publication: *SHELXL97*.

## Supplementary Material

Crystal structure: contains datablock(s) I, global. DOI: 10.1107/S160053681204411X/lh5541sup1.cif

Structure factors: contains datablock(s) I. DOI: 10.1107/S160053681204411X/lh5541Isup2.hkl

Additional supplementary materials:  crystallographic information; 3D view; checkCIF report

## Figures and Tables

**Table 1:** Hydrogen-bond geometry (Å, °)

*D*—H⋯*A*	*D*—H	H⋯*A*	*D*⋯*A*	*D*—H⋯*A*
O1—H1*A*⋯O16^i^	0.83 (2)	1.98 (2)	2.805 (3)	175 (3)
O1—H1*B*⋯O17^ii^	0.82 (3)	1.81 (3)	2.631 (3)	175 (4)
O2—H2*A*⋯O16^iii^	0.83 (4)	2.04 (4)	2.840 (3)	162 (4)
O2—H2*B*⋯O17^iv^	0.83 (3)	1.94 (3)	2.767 (3)	173 (3)

Symmetry codes: (i) 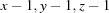; (ii) 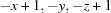; (iii) 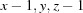; (iv) 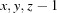.

**Fig. 1.**
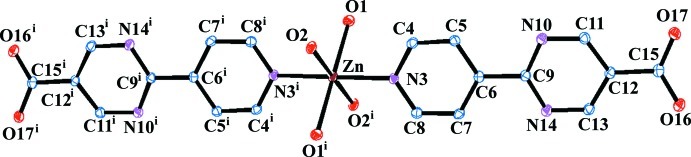
The molecular structure of the title compound. Displacement ellipsoids are drawn at the 30% probability level [Symmetry code: (i) -x,-y, -z].

**Fig. 2.**
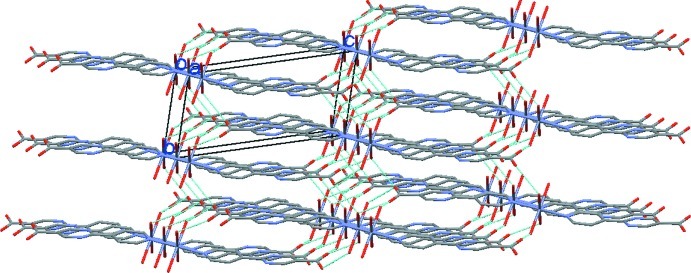
Part of the crystal structure showing the three-dimensional hydrogen-bonded network.

## supplementary crystallographic information

### Comment

The aim of designing coordination frameworks is now been motivated through the field of supramolecular chemistry (Collet *et al.*, 1996) and crystal engineering (Sen *et al.*, 2012) from the viewpoints of the development of novel multi-functional MOFs. Fabricating MOFs with the desired properties are now the present day challenges of the chemist. N-Heterocyclic carboxylic acids share a major role in developing MOFs based material synthesis (Sen *et al.*, 2012, Saha *et al.* 2012). Herein, we wish to report a new compound having an N-heterocyclic carboxylate ligand (2-pyridin-4-ylpyrimidine-5-carboxylato).

The molecular structure of the title compound is shown in Fig. 1. The Zn^II^ ion lies on an inversion center and is coordinated in a slightly distorted octahedral geometry by two N atoms from two 2-pyridin-4-ylpyrimidine-5-carboxylato ligands and four water molecules. The equatorial plane is formed by the four water molecule and the two axial sites are occupied by the N-donor sites of the ligand. In the crystal, O—H···O hydrogen bonds form a three-dimensional network (Fig. 2). The related structure, tetraaquabis[4-(4H-1,2,4-triazol-4-yl)benzoato- κN^1^]manganese(II) decahydrate, has been published (Piao & Xuan, 2011).

### Experimental

To prepare the complex we followed a routine hydrothermal process. Zn(NO_3_)_2_ hydrate and 2-pyridin-4-ylpyrimidine-5-carboxylic acid were mixed in a 1:1 ratio, and kept in a reaction bomb at 433 K for 2 days in autogenously created pressure. After cooling to room temperature colourless block-shaped crystals were obtained. Yield *ca*. 45% (based on metal). The crystals were collected by filtration, washed thoroughly with water and dried in ambient conditions.

### Refinement

The hydrogen atoms of the C—H bonds were placed at calculated positions and refined as riding atoms, with C—H = 0.95 Å with *U*_iso_(H) =1.2U_eq_(C) of the atom to which they are attached. The positions of the hydrogen atoms of the water molecules were discernible in difference Fourier maps and they were included in the structure refinement with individual isotropic thermal parameters and refined with an O—H distance restraint of 0.83 (2) Å.

### Crystal data

**Table:**

[Zn(C_10_H_6_N_3_O_2_)_2_(H_2_O)_4_]	*Z* = 1
*M**_r_* = 537.79	*F*(000) = 276
Triclinic, *P*1	*D*_x_ = 1.781 Mg m^−^^3^
Hall symbol: -P 1	Mo *K*α radiation, λ = 0.71073 Å
*a* = 6.2764 (7) Å	Cell parameters from 246 reflections
*b* = 6.9208 (7) Å	θ = 2.6–27.6°
*c* = 12.7810 (17) Å	µ = 1.29 mm^−^^1^
α = 99.676 (7)°	*T* = 150 K
β = 92.638 (7)°	Block, colourless
γ = 112.639 (5)°	0.26 × 0.20 × 0.04 mm
*V* = 501.36 (10) Å^3^	

### Data collection

**Table:**

Bruker SMART CCD diffractometer	2184 independent reflections
Radiation source: fine-focus sealed tube	2030 reflections with *I* > 2σ(*I*)
Graphite monochromator	*R*_int_ = 0.032
fine–focus sealed tube scans	θ_max_ = 27.4°, θ_min_ = 3.3°
Absorption correction: multi-scan (*SADABS*; Bruker, 2008)	*h* = −8→8
*T*_min_ = 0.730, *T*_max_ = 0.950	*k* = −8→8
8143 measured reflections	*l* = −16→16

### Refinement

**Table:**

Refinement on *F*^2^	Primary atom site location: structure-invariant direct methods
Least-squares matrix: full	Secondary atom site location: difference Fourier map
*R*[*F*^2^ > 2σ(*F*^2^)] = 0.033	Hydrogen site location: inferred from neighbouring sites
*wR*(*F*^2^) = 0.089	H atoms treated by a mixture of independent and constrained refinement
*S* = 1.14	*w* = 1/[σ^2^(*F*_o_^2^) + (0.0522*P*)^2^ + 0.1478*P*] where *P* = (*F*_o_^2^ + 2*F*_c_^2^)/3
2184 reflections	(Δ/σ)_max_ < 0.001
176 parameters	Δρ_max_ = 1.05 e Å^−^^3^
4 restraints	Δρ_min_ = −0.61 e Å^−^^3^

### Special details

**Table:**

Geometry. All e.s.d.'s (except the e.s.d. in the dihedral angle between two l.s. planes) are estimated using the full covariance matrix. The cell e.s.d.'s are taken into account individually in the estimation of e.s.d.'s in distances, angles and torsion angles; correlations between e.s.d.'s in cell parameters are only used when they are defined by crystal symmetry. An approximate (isotropic) treatment of cell e.s.d.'s is used for estimating e.s.d.'s involving l.s. planes.
Refinement. Refinement of *F*^2^ against ALL reflections. The weighted *R*-factor *wR* and goodness of fit *S* are based on *F*^2^, conventional *R*-factors *R* are based on *F*, with *F* set to zero for negative *F*^2^. The threshold expression of *F*^2^ > σ(*F*^2^) is used only for calculating *R*-factors(gt) *etc*. and is not relevant to the choice of reflections for refinement. *R*-factors based on *F*^2^ are statistically about twice as large as those based on *F*, and *R*- factors based on ALL data will be even larger.

### Fractional atomic coordinates and isotropic or equivalent isotropic displacement parameters (Å^2^)

**Table:**

	*x*	*y*	*z*	*U*_iso_*/*U*_eq_	
Zn	0.0000	0.0000	0.0000	0.01467 (13)	
O1	0.1899 (3)	−0.1897 (2)	−0.03156 (12)	0.0170 (3)	
H1A	0.129 (5)	−0.303 (3)	−0.0767 (17)	0.028 (7)*	
H1B	0.238 (6)	−0.227 (6)	0.0190 (19)	0.051 (10)*	
O2	0.2499 (3)	0.2665 (3)	−0.05357 (13)	0.0196 (3)	
H2A	0.184 (5)	0.334 (5)	−0.080 (2)	0.044 (9)*	
H2B	0.365 (4)	0.265 (5)	−0.082 (2)	0.046 (10)*	
N3	0.1681 (3)	0.1214 (3)	0.16096 (14)	0.0152 (4)	
C4	0.0418 (4)	0.1047 (3)	0.24398 (17)	0.0165 (4)	
H4	−0.1216	0.0610	0.2296	0.020*	
C5	0.1386 (4)	0.1481 (3)	0.34911 (16)	0.0159 (4)	
H5	0.0430	0.1334	0.4053	0.019*	
C6	0.3782 (4)	0.2137 (3)	0.37137 (16)	0.0138 (4)	
C7	0.5107 (4)	0.2394 (3)	0.28616 (17)	0.0167 (4)	
H7	0.6751	0.2890	0.2985	0.020*	
C8	0.3995 (4)	0.1916 (3)	0.18334 (17)	0.0173 (4)	
H8	0.4914	0.2094	0.1259	0.021*	
C9	0.4895 (4)	0.2521 (3)	0.48227 (16)	0.0148 (4)	
N10	0.3511 (3)	0.2432 (3)	0.56043 (14)	0.0167 (4)	
C11	0.4536 (4)	0.2753 (3)	0.65945 (17)	0.0169 (4)	
H11	0.3620	0.2685	0.7169	0.020*	
C12	0.6871 (4)	0.3180 (3)	0.68231 (16)	0.0146 (4)	
C13	0.8139 (4)	0.3264 (4)	0.59538 (17)	0.0179 (4)	
H13	0.9751	0.3571	0.6078	0.022*	
N14	0.7165 (3)	0.2928 (3)	0.49487 (14)	0.0179 (4)	
C15	0.7950 (4)	0.3495 (3)	0.79525 (16)	0.0166 (4)	
O16	1.0133 (3)	0.4300 (3)	0.81382 (12)	0.0203 (3)	
O17	0.6557 (3)	0.2901 (3)	0.86250 (12)	0.0214 (3)	

### Atomic displacement parameters (Å^2^)

**Table:**

	*U*^11^	*U*^22^	*U*^33^	*U*^12^	*U*^13^	*U*^23^
Zn	0.01318 (19)	0.0201 (2)	0.01366 (19)	0.00978 (14)	0.00064 (12)	0.00383 (13)
O1	0.0168 (7)	0.0215 (8)	0.0163 (8)	0.0118 (7)	−0.0000 (6)	0.0033 (6)
O2	0.0167 (8)	0.0232 (9)	0.0225 (8)	0.0101 (7)	0.0040 (6)	0.0086 (6)
N3	0.0151 (8)	0.0197 (9)	0.0140 (8)	0.0103 (7)	0.0014 (7)	0.0033 (7)
C4	0.0140 (10)	0.0187 (11)	0.0192 (10)	0.0090 (8)	0.0020 (8)	0.0038 (8)
C5	0.0160 (10)	0.0186 (11)	0.0160 (10)	0.0091 (9)	0.0040 (8)	0.0048 (8)
C6	0.0168 (10)	0.0114 (10)	0.0157 (10)	0.0080 (8)	0.0011 (8)	0.0036 (7)
C7	0.0132 (10)	0.0192 (11)	0.0198 (11)	0.0088 (9)	0.0016 (8)	0.0036 (8)
C8	0.0161 (10)	0.0213 (11)	0.0163 (10)	0.0092 (9)	0.0035 (8)	0.0038 (8)
C9	0.0162 (10)	0.0140 (10)	0.0166 (10)	0.0079 (8)	0.0014 (8)	0.0044 (8)
N10	0.0160 (9)	0.0193 (9)	0.0170 (9)	0.0089 (7)	0.0017 (7)	0.0051 (7)
C11	0.0185 (10)	0.0178 (11)	0.0161 (10)	0.0084 (9)	0.0022 (8)	0.0051 (8)
C12	0.0161 (10)	0.0130 (10)	0.0174 (10)	0.0082 (8)	0.0004 (8)	0.0046 (8)
C13	0.0158 (10)	0.0213 (11)	0.0190 (11)	0.0100 (9)	0.0006 (8)	0.0045 (8)
N14	0.0159 (9)	0.0225 (10)	0.0170 (9)	0.0094 (8)	0.0008 (7)	0.0045 (7)
C15	0.0201 (10)	0.0160 (11)	0.0177 (10)	0.0118 (9)	0.0008 (8)	0.0030 (8)
O16	0.0170 (7)	0.0255 (8)	0.0198 (8)	0.0100 (7)	−0.0008 (6)	0.0053 (6)
O17	0.0199 (8)	0.0330 (9)	0.0179 (8)	0.0156 (7)	0.0040 (6)	0.0096 (6)

### Geometric parameters (Å, º)

**Table:**

Zn—O1	2.0923 (15)	C6—C7	1.394 (3)
Zn—O1^i^	2.0923 (15)	C6—C9	1.486 (3)
Zn—N3^i^	2.1419 (17)	C7—C8	1.384 (3)
Zn—N3	2.1420 (17)	C7—H7	0.9500
Zn—O2^i^	2.1512 (15)	C8—H8	0.9500
Zn—O2	2.1512 (15)	C9—N14	1.338 (3)
O1—H1A	0.830 (10)	C9—N10	1.348 (3)
O1—H1B	0.822 (10)	N10—C11	1.336 (3)
O2—H2A	0.833 (10)	C11—C12	1.385 (3)
O2—H2B	0.827 (10)	C11—H11	0.9500
N3—C8	1.341 (3)	C12—C13	1.392 (3)
N3—C4	1.347 (3)	C12—C15	1.510 (3)
C4—C5	1.383 (3)	C13—N14	1.340 (3)
C4—H4	0.9500	C13—H13	0.9500
C5—C6	1.393 (3)	C15—O16	1.257 (3)
C5—H5	0.9500	C15—O17	1.258 (3)
			
O1—Zn—O1^i^	180.0	C4—C5—H5	120.5
O1—Zn—N3^i^	88.59 (6)	C6—C5—H5	120.5
O1^i^—Zn—N3^i^	91.41 (6)	C5—C6—C7	118.01 (19)
O1—Zn—N3	91.41 (6)	C5—C6—C9	121.15 (18)
O1^i^—Zn—N3	88.59 (6)	C7—C6—C9	120.83 (19)
N3^i^—Zn—N3	180.0	C8—C7—C6	119.2 (2)
O1—Zn—O2^i^	86.31 (6)	C8—C7—H7	120.4
O1^i^—Zn—O2^i^	93.69 (6)	C6—C7—H7	120.4
N3^i^—Zn—O2^i^	91.45 (6)	N3—C8—C7	123.13 (19)
N3—Zn—O2^i^	88.55 (6)	N3—C8—H8	118.4
O1—Zn—O2	93.69 (6)	C7—C8—H8	118.4
O1^i^—Zn—O2	86.31 (6)	N14—C9—N10	126.45 (19)
N3^i^—Zn—O2	88.55 (6)	N14—C9—C6	117.08 (18)
N3—Zn—O2	91.45 (6)	N10—C9—C6	116.48 (19)
O2^i^—Zn—O2	180.0	C11—N10—C9	115.59 (19)
Zn—O1—H1A	118 (2)	N10—C11—C12	123.16 (19)
Zn—O1—H1B	118 (2)	N10—C11—H11	118.4
H1A—O1—H1B	103 (3)	C12—C11—H11	118.4
Zn—O2—H2A	110 (2)	C11—C12—C13	116.22 (19)
Zn—O2—H2B	125 (2)	C11—C12—C15	121.41 (18)
H2A—O2—H2B	114 (3)	C13—C12—C15	122.36 (19)
C8—N3—C4	117.43 (18)	N14—C13—C12	122.3 (2)
C8—N3—Zn	121.52 (14)	N14—C13—H13	118.8
C4—N3—Zn	120.54 (14)	C12—C13—H13	118.8
N3—C4—C5	123.19 (19)	C9—N14—C13	116.28 (18)
N3—C4—H4	118.4	O16—C15—O17	125.88 (19)
C5—C4—H4	118.4	O16—C15—C12	117.94 (18)
C4—C5—C6	118.99 (19)	O17—C15—C12	116.18 (19)

Symmetry code: (i) −*x*, −*y*, −*z*.

### Hydrogen-bond geometry (Å, º)

**Table:**

*D*—H···*A*	*D*—H	H···*A*	*D*···*A*	*D*—H···*A*
O1—H1*A*···O16^ii^	0.83 (2)	1.98 (2)	2.805 (3)	175 (3)
O1—H1*B*···O17^iii^	0.82 (3)	1.81 (3)	2.631 (3)	175 (4)
O2—H2*A*···O16^iv^	0.83 (4)	2.04 (4)	2.840 (3)	162 (4)
O2—H2*B*···O17^v^	0.83 (3)	1.94 (3)	2.767 (3)	173 (3)

Symmetry codes: (ii) *x*−1, *y*−1, *z*−1; (iii) −*x*+1, −*y*, −*z*+1; (iv) *x*−1, *y*, *z*−1; (v) *x*, *y*, *z*−1.
